# Screening potential prognostic biomarkers of long non-coding RNAs for predicting the risk of chronic kidney disease

**DOI:** 10.1590/1414-431X20198333

**Published:** 2019-11-07

**Authors:** Na Li, Yan Cui, Min Yin, Feng Liu

**Affiliations:** 1 Department of Nephrology, China-Japan Union Hospital of Jilin University, Changchun, Jilin Province, China; 2 Department of Nephrology, The First Hospital of Jilin University, Changchun, Jilin Province, China

**Keywords:** Long non-coding RNAs, Chronic kidney disease, WGCNA, Prognostic biomarkers

## Abstract

Not much is known about the roles of long non-coding RNAs (lncRNAs) for chronic kidney disease (CKD). In this study, we included CKD patient cohorts and normal controls as a discovery cohort to identify putative lncRNA biomarkers associated with CKD. We first compared the lncRNA expression profiles of CKD patients with normal controls, and identified differentially expressed lncRNAs and mRNAs. Co-expression network based on the enriched differentially expressed mRNAs and lncRNAs was constructed using WGCNA to identify important modules related to CKD. A lncRNA-miRNA-mRNA pathway network based on the hub lncRNAs and mRNAs, related miRNAs, and overlapping pathways was further constructed to reveal putative biomarkers. A total of 821 significantly differentially expressed mRNAs and lncRNAs were screened between CKD and control samples, which were enriched in nine modules using weighted correlation network analysis (WGCNA), especially brown and yellow modules. Co-expression network based on the enriched differentially expressed mRNAs and lncRNAs in brown and yellow modules uncovered 7 hub lncRNAs and 53 hub mRNAs. A lncRNA-miRNA-mRNA pathway network further revealed that lncRNAs of *HCP5* and *NOP14-AS1* and genes of *CCND2*, *COL3A1*, *COL4A1*, and *RAC2* were significantly correlated with CKD. The lncRNAs of *NOP14-AS1* and *HCP5* were potential prognostic biomarkers for predicting the risk of CKD.

## Introduction

Arising from many heterogeneous disease pathways, chronic kidney disease (CKD) can alter the function and structure of the kidney irreversibly, over months or years. In 2012, according to World Health Organization global health estimates, 864 226 deaths (or 1.5% of deaths worldwide) were attributable to CKD. Thus, the burden of CKD is substantial. The etiology of CKD is very complex, with hypertension and diabetes being the main causes. Moreover, increased risk of developing CKD and more rapidly progressing CKD are reported to be related to worsening blood pressure control. Recently, investigation of people with genetic causes of CKD has been evolving rapidly. Several loci, genetic polymorphisms, and single nucleotide polymorphisms that might contribute to accelerated progression of CKD have been identified with genome-wide association studies (1–4).

During past years, the biological roles of various types of non-coding RNAs (ncRNAs) have been highlighted. Long non-coding RNAs (lncRNAs), a newly discovered class of ncRNAs, were defined as RNA molecules longer than 200 nucleotides in length. There is growing evidence that lncRNAs are involved in various biological processes, including maintenance of pluripotency, nuclear organization, development, translational control, and RNA splicing. A growing number of lncRNAs have been characterized as tumor suppressor genes or oncogenes contributing to cancer development, progression, and metastasis (3,5,6). Nevertheless, not much is known about the roles of lncRNAs for chronic human diseases other than cancer until recently, such as CKD.

In this work, CKD patient cohorts and normal controls were included as a discovery cohort to identify putative lncRNA biomarkers. The lncRNA expression profiles of CKD patients were compared with normal controls, and differentially expressed lncRNAs and mRNAs were identified. Co-expression network based on the enriched differentially expressed mRNAs and lncRNAs was constructed using weighted correlation network analysis (WGCNA) to identify important modules related to CKD. A lncRNA-miRNA-mRNA-pathway network based on the hub lncRNAs and mRNAs, related miRNAs, and overlapping pathways was further constructed to reveal putative biomarkers for CKD.

## Material and Methods

### Microarray data and data preprocessing

The microarray data GSE48944 for CKD was downloaded from the National Center for Biotechnology Information (NCBI) Gene Expression Omnibus database (GEO) on May 7, 2018 (7). The testing platform was Affymetrix Human Genome U133A 2.0 (GPL571: HG-U133A_2). GSE48944 included 13 human CKD samples and 12 control (CTRL) samples. Microarray raw data (.CEL files) of the CKD were processed with oligo Version 1.41.1 (http://www.bioconductor.org/packages/release/bioc/html/oligo.html) in R language to achieve an approximate normal distribution (8). Subsequently, the data were standardized using the median normalization and quantile methods.

### Screening of differentially expressed mRNAs and lncRNAs

The probe sequences (GPL571) of Affymetrix HG-U133_2 array were downloaded from the Affymetrix website (https://www.ncbi.nlm.nih.gov/geo/query/acc.cgi?acc=GPL571). Along with information of transcript ID, RefSeq ID, and chromosomal position, the 3909 lncRNAs and 19198 protein coding genes in HUGO Gene Nomenclature Committee (HGNC, http://www.genenames.org/) were used to re-annotate the mRNAs and lncRNAs in the microarray data (9). Limma Version 3.34.0 (https://bioconductor.org/packages/release/bioc/html/limma.html) in R language (R3.4.1) was used to determine the false discovery rate (FDR) and fold changes (FC) of differentially expressed mRNAs and lncRNAs (10). The FDR value <0.05 and |logFC|>0.5 were used as the cut-off criteria. Based on expression profiles of mRNAs and lncRNAs, the pheatmap Version 1.0.8 (https://cran.r-project.org/package=pheatmap) in R language (R3.4.1) was used to determine the metric of Euclidean distance and bilateral hierarchical clustering, and displayed with hierarchical clustering (11–13). Functional enrichment analysis for lncRNAs was conducted using DAVID Bioinformatics Tool (DAVID 6.8, https://david.ncifcrf.gov/) limited to Gene Ontology (GO) terms and Kyoto Encyclopedia of Genes and Genomes (KEGG) categories to screen the significantly enriched GO functions and KEGG pathways (14,15).

### WGCNA analysis to screen specific modules and RNAs

WGCNA, a biology systems method, can be used to construct a scale-free network from gene expression data. WGCNA package Version 1.61 in R (https://cran.r-project.org/web/packages/WGCNA/) was used to screen specific modules and RNAs related to CKD (16–18). The modules were detected using dynamic tree cut algorithm with a minimum module size of 100 and a minimum cut height of 0.95 (P value <0.05). The significantly differentially expressed mRNAs and lncRNAs were mapped to modules in WGCNA. Fold enrichments and P values of significantly differentially expressed mRNAs and lncRNAs in modules were calculated using the hypergeometric test of f(k,N,M,n) = C(k,M) * C(n-k,N-M) / C(n,N) (19). A fold enrichment >1 and P value <0.05 were used as the cut-off criteria.

### Network construction of enriched differentially expressed mRNAs and lncRNAs

Pearson’s correlation coefficient (PCC) of the enriched differentially expressed mRNAs and lncRNAs in WGCNA modules was calculated using the cor function in R (http://77.66.12.57/R-help/cor.test.html), and the co-expression network was constructed. Cytoscape 3.6.1 (http://www.cytoscape.org/) was used to visualize the co-expression network of enriched differentially expressed mRNAs and lncRNAs in WGCNA modules (20). GO functional and KEGG pathway analyses for enriched differentially expressed mRNAs and lncRNAs in the co-expression network were searched using the Database for Annotation, Visualization, and Integrated Discovery (DAVID, http://www.david.niaid.nih.gov) software. Three categories of biological process, cellular compartment, and molecular function were included in the GO terms.

### Construction of KEGG pathway network related to CKD

KEGG pathways related to CKD were searched with Comparative Toxic Genomics Database 2017 update (CTD, http://ctd.mdibl.org/) using the keyword “Chronic Kidney Disease”. These pathways were compared with KEGG pathways enriched in the co-expression network of differentially expressed mRNAs and lncRNAs, and then the overlapping pathways were obtained. A KEGG pathway network related to CKD was constructed using these overlapping pathways. We searched the related miRNAs using StarBase Version 2.0 database (http://starbase.sysu.edu.cn/), constructed lncRNA-miRNA-mRNA-pathway network by connecting the hubs, and screened the potential prognostic biomarkers of long non-coding RNAs for predicting the risk of CKD (21).

## Results

### Overview of differential expression analysis (lncRNAs and mRNAs)

**Figure 1.**
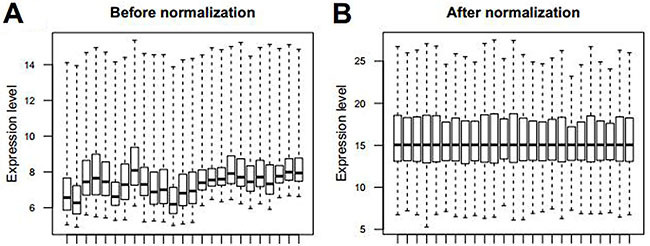
Boxplot of microarray raw data before (**A**) and after (**B**) normalization.

Firstly, the microarray raw data were standardized using the median normalization and quantile methods. Boxplots of microarray raw data before and after normalization are shown in Figure 1.

**Figure 2.**
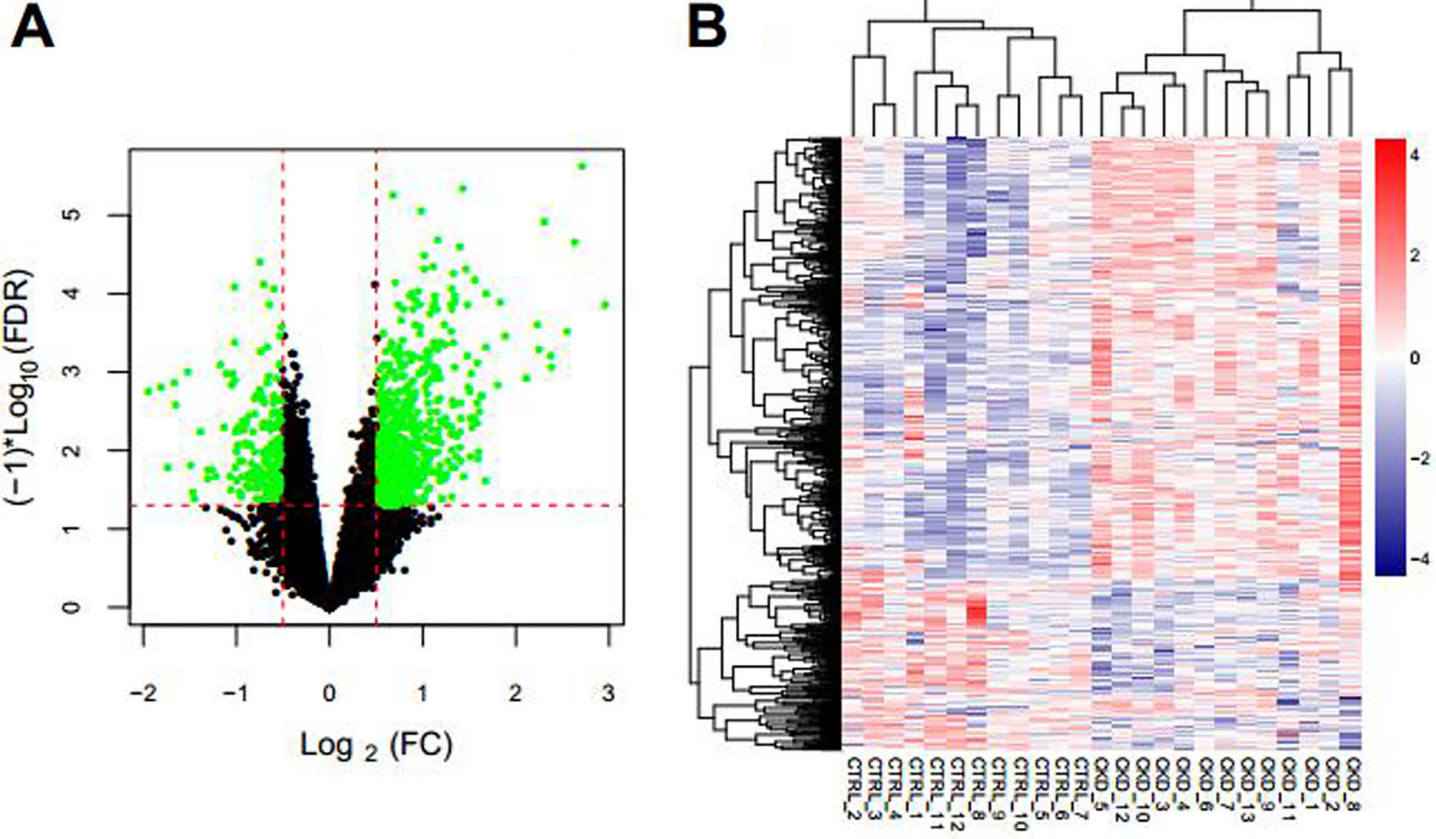
**A**, Volcano plot of log_2_ fold change (FC) – log_10_ false discovery rate (FDR) test for differentially expressed mRNAs and lncRNAs. Red dotted line indicates FDR <0.05. Green dots indicate differentially expressed mRNAs and lncRNAs. **B**, Heatmap of hierarchical clustering of differentially expressed lncRNAs and mRNAs.

Next, we performed differentially expressed analysis to identify potential lncRNAs associated with CKD. A total of 130 lncRNAs and 12,126 protein-coding genes were obtained after re-annotation, as shown in Table S1. A total of 821 significantly differentially expressed mRNAs and lncRNAs were screened using Limma (http://www.bioconductor.org/packages/release/bioc/html/limma.html), including 205 downregulated and 616 upregulated between CKD and CTRL (Figure 2A and Table S2). The expression level of these significantly differentially expressed mRNAs and lncRNAs are shown in Table S3. Bilateral hierarchical clustering of significantly differentially expressed mRNAs and lncRNAs are shown in Figure 2B. The expression level of RNAs could discriminate CKD and CTRL samples, indicating distinctive features between these samples.

**Figure 3.**
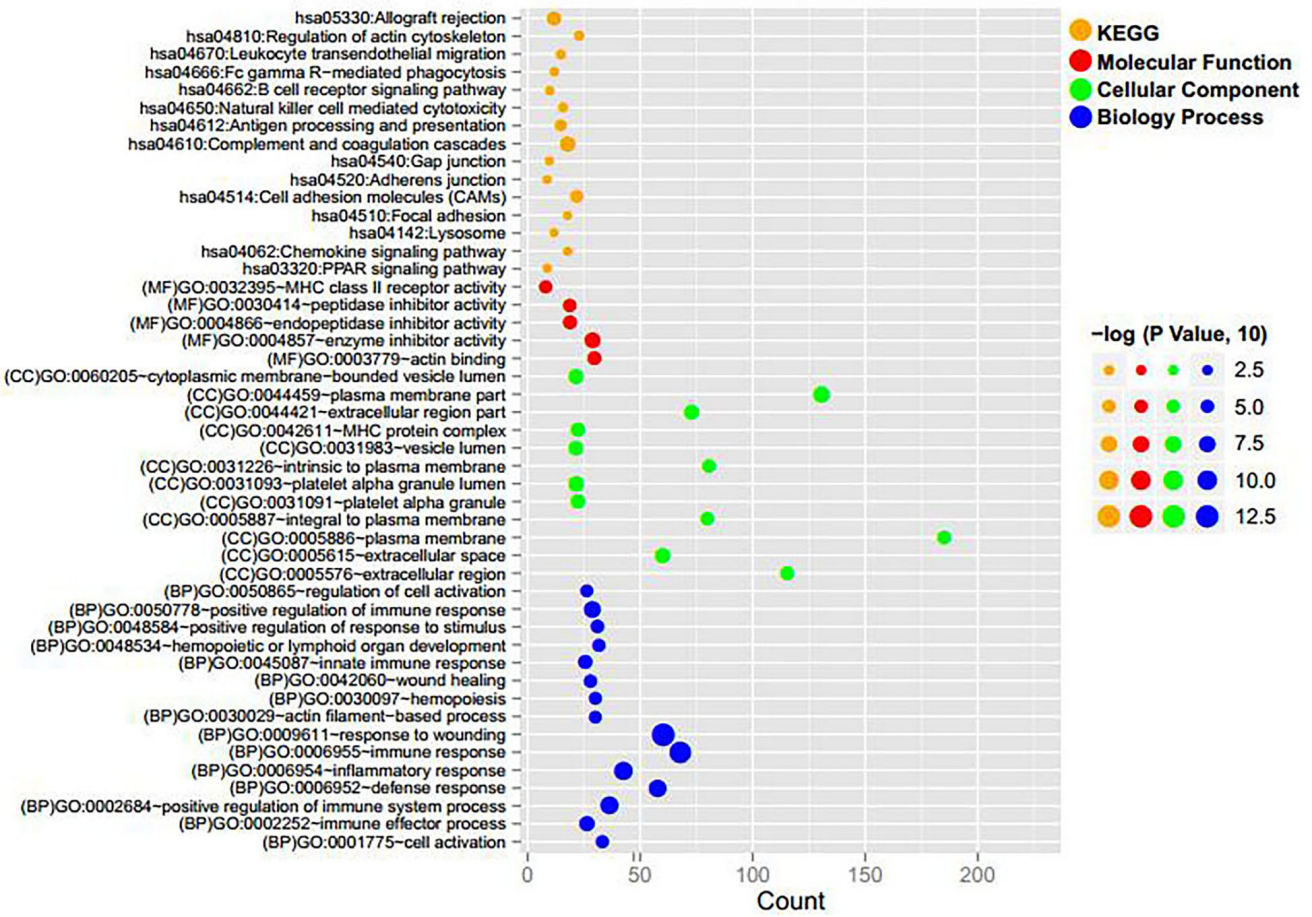
Distribution diagram of enriched Gene Ontology (GO) functions and KEGG pathways of differentially expressed mRNAs and lncRNAs. Orange, red, green, and blue dots indicate KEGG pathway, molecular function, cellular component, and biological process, respectively.

GO functional and KEGG pathway analyses for 802 differentially expressed coding RNAs indicated that 32 GO functions (15 biological processes, 12 cellular compartments, and 5 molecular functions) and 15 KEGG pathways were enriched. As shown in Table 1 and Figure 3, these differentially expressed coding RNAs were related to GO functions of wound response, immune response, and inflammatory response, as well as KEGG pathways of complement and coagulation cascades (hsa04610), allograft rejection (hsa05330), and cell adhesion molecules (hsa04514), etc.

**Table 1.** Enriched GO functions and KEGG pathways of differentially expressed coding RNAs.

Category	Term	Count	P value
Biology Process	GO: 0009611∼response to wounding	67	2.55E-13
	GO: 0006955∼immune response	77	2.08E-12
	GO: 0006954∼inflammatory response	44	6.85E-10
	GO: 0002684∼positive regulation of immune system process	36	1.67E-09
	GO: 0006952∼defense response	64	3.54E-09
	GO: 0050778∼positive regulation of immune response	26	1.47E-08
	GO: 0002252∼immune effector process	23	2.63E-07
	GO: 0045087∼innate immune response	22	1.77E-06
	GO: 0048584∼positive regulation of response to stimulus	29	5.96E-06
	GO: 0042060∼wound healing	25	1.04E-05
	GO: 0001775∼cell activation	32	1.33E-05
	GO: 0048534∼hemopoietic or lymphoid organ development	30	1.36E-05
	GO: 0030097∼hemopoiesis	28	1.70E-05
	GO: 0050865∼regulation of cell activation	23	2.39E-05
	GO: 0030029∼actin filament-based process	28	2.49E-05
Cellular Component	GO: 0044459∼plasma membrane part	155	2.53E-08
	GO: 0031093∼platelet alpha granule lumen	13	2.00E-07
	GO: 0005615∼extracellular space	63	2.65E-07
	GO: 0044421∼extracellular region part	80	3.03E-07
	GO: 0060205∼cytoplasmic membrane-bounded vesicle lumen	13	4.71E-07
	GO: 0031983∼vesicle lumen	13	7.98E-07
	GO: 0031091∼platelet alpha granule	14	1.19E-06
	GO: 0042611∼MHC protein complex	14	1.48E-06
	GO: 0005886∼plasma membrane	226	3.93E-06
	GO: 0005576∼extracellular region	135	4.08E-06
	GO: 0005887∼integral to plasma membrane	89	5.27E-06
	GO: 0031226∼intrinsic to plasma membrane	90	7.08E-06
Molecular Function	GO: 0004857∼enzyme inhibitor activity	35	1.04E-07
	GO: 0003779∼actin binding	36	3.10E-06
	GO: 0004866∼endopeptidase inhibitor activity	22	3.18E-06
	GO: 0030414∼peptidase inhibitor activity	22	7.56E-06
	GO: 0032395∼MHC class II receptor activity	8	1.36E-05
KEGG Pathway	hsa04610: Complement and coagulation cascades	18	2.04E-07
	hsa05330: Allograft rejection	12	2.50E-06
	hsa04514: Cell adhesion molecules (CAMs)	22	1.69E-05
	hsa04612: Antigen processing and presentation	15	0.000189
	hsa04810: Regulation of actin cytoskeleton	23	0.004886
	hsa04670: Leukocyte transendothelial migration	15	0.00551
	hsa04650: Natural killer cell mediated cytotoxicity	16	0.006762
	hsa04666: Fc gamma R-mediated phagocytosis	12	0.013475
	hsa04662: B cell receptor signaling pathway	10	0.017701
	hsa03320: PPAR signaling pathway	9	0.027244
	hsa04062: Chemokine signaling pathway	18	0.028853
	hsa04540: Gap junction	10	0.043395
	hsa04520: Adherens junction	9	0.04559
	hsa04142: Lysosome	12	0.046965
	hsa04510: Focal adhesion	18	0.048777

GO: Gene Ontology.

### Identification and characterization of CKD-associated modules using WGCNA

**Figure 4.**
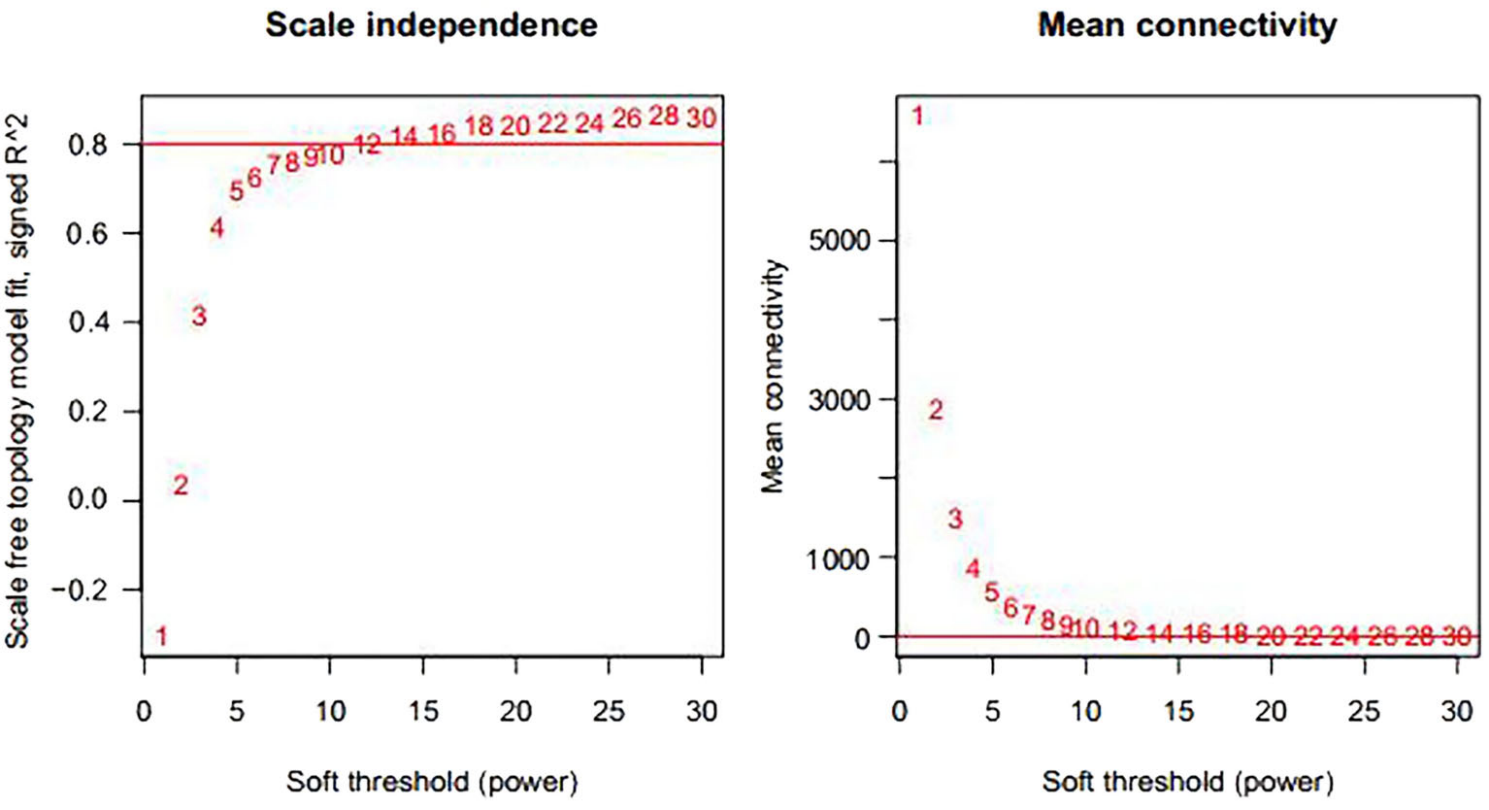
**Left panel**: power selection map. **Right panel**: mean connectivity degree of RNA under different power values.

**Figure 5.**
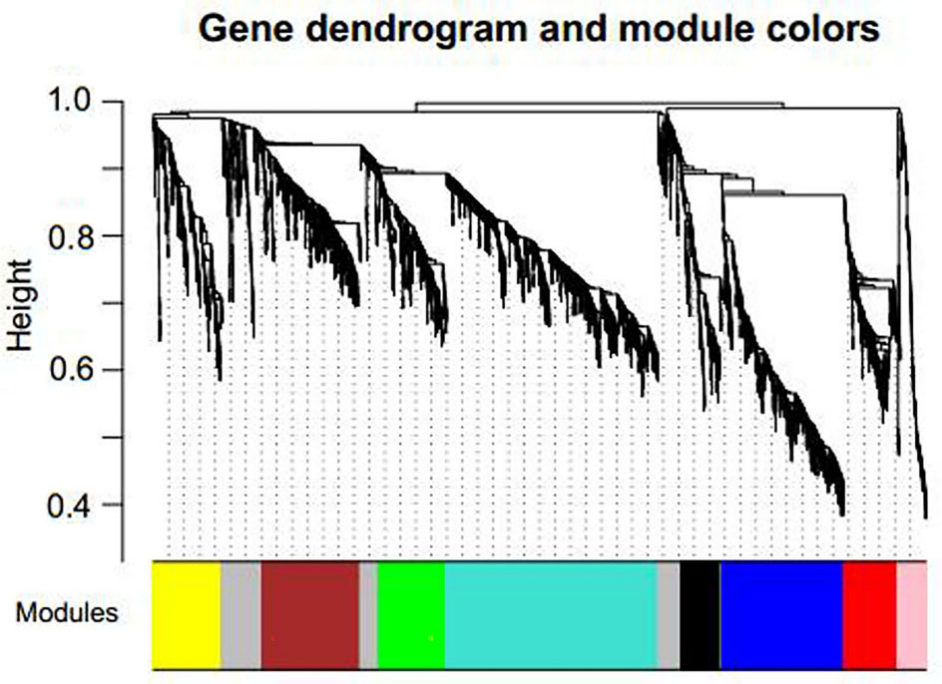
Network construction and module identification.

The underlying molecular mechanisms of CKD could be explored through an approach of gene co-expression network. It allows us to explore a set of interacting mRNAs and lncRNAs measured by modules or subnetworks that are involved in the complex disease of CKD. The WGCNA R package implements a suite of tools, which can be used for the network construction. A weighted adjacency matrix implemented in WGCNA was used to construct a scale-free network and identify important modules related to CKD. In this work, a step-by-step network construction and module detection method was used, and a selected power (power=12) was determined through a soft-threshold approach implemented in WGCNA, as shown in Figure 4. Co-expression modules were defined by a robust dynamic hierarchical tree and sets of tightly co-regulated genes with the measurement of dissimilarity. The minimum module size of 100 and a minimum cut height of 0.25 were set to ensure a qualified number of genes for the further analysis. Nine co-expression modules were obtained by clustering the highly co-expressed genes in the constructed network (Table 2). With each clustered module showing a different color, they were visualized as shown in Figure 5. Differently expressed mRNAs and lncRNAs were significantly enriched in brown and yellow modules, including 151 and 54 RNAs (containing 7 lncRNAs), respectively. These 205 differently expressed mRNAs and lncRNAs enriched in brown and yellow modules were used for subsequent investigation. The list of these RNAs in brown and yellow modules are provided in Table S4.

**Table 2.** Summary of nine modules in co-expression network.

Color	RNAs (#)	Correlation	P_corr_	DE RNAs (n)	Enrichment fold (95% CI)	P_hyper_
Black	171	0.832	0.000338	1	0.0615 (0.00155-0.350)	6.75E-06
Blue	519	0.863	0.000936	5	0.101 (0.0325-0.241)	1.35E-13
Brown	421	0.861	0.00209	151	3.771 (3.006-4.716)	2.20E-16
Green	289	0.819	0.000286	17	0.619 (0.351-1.025)	0.526
Grey	355	0.129	0.282	22	0.652 (0.397-1.021)	6.34E-02
Pink	122	0.724	0.0263	1	0.0862 (0.00216-0.493)	3.87E-04
Red	229	0.861	0.000317	1	0.0459 (0.00116-0.261)	7.07E-08
Turquoise	903	0.794	0.00102	62	0.722 (0.5354-0.9611)	2.46E-02
Yellow	293	0.889	0.0266	54	1.938 (1.389-2.664)	9.10E-05

DE: differently expressed.

### Network analysis revealed prognostic lncRNA biomarkers associated with CKD progression

**Figure 6.**
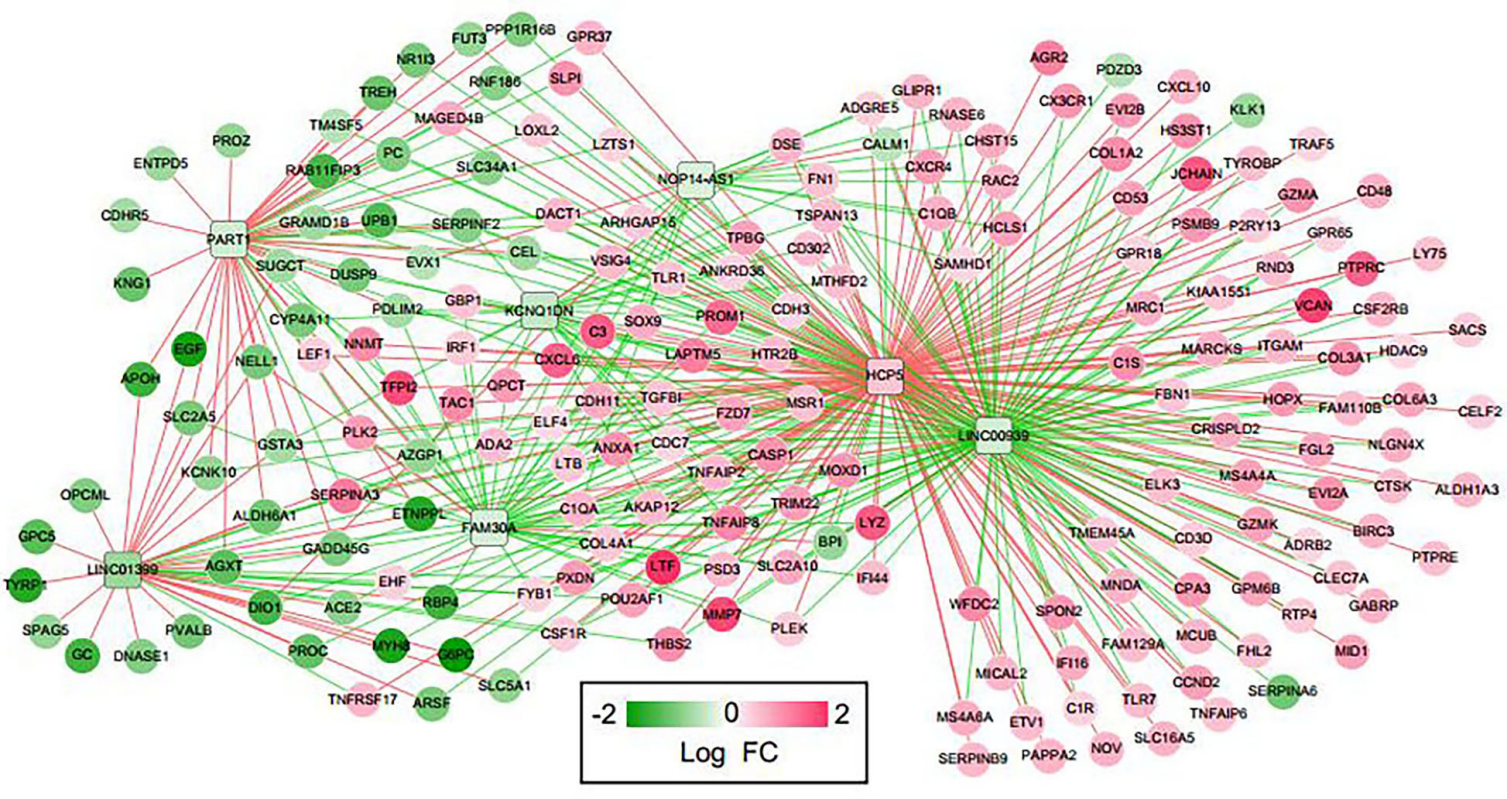
lncRNA-mRNA co-expression network. Green lines represent negative correlations, and pink lines represent positive correlations. Squares indicate lncRNA, and circles indicate mRNA. The node change from green to red indicates that log fold change (FC) changed from negative to positive.

To identify potential prognostic lncRNA biomarkers associated with CKD progression, we further constructed a co-expression network based on the enriched differentially expressed mRNAs and lncRNAs in brown and yellow modules. PCC of 197 differentially expressed mRNAs and 7 differentially expressed lncRNAs were calculated (Table S5). As shown in Figure 6, lncRNA-mRNA co-expression network contained 462 lines (250 with negative correlation and 212 with positive correlation) and 204 hubs. Among these 7 lncRNAs, 1 lncRNA was upregulated and 6 lncRNAs were downregulated, while 53 mRNAs were downregulated and 144 mRNAs were upregulated.

**Figure 7.**
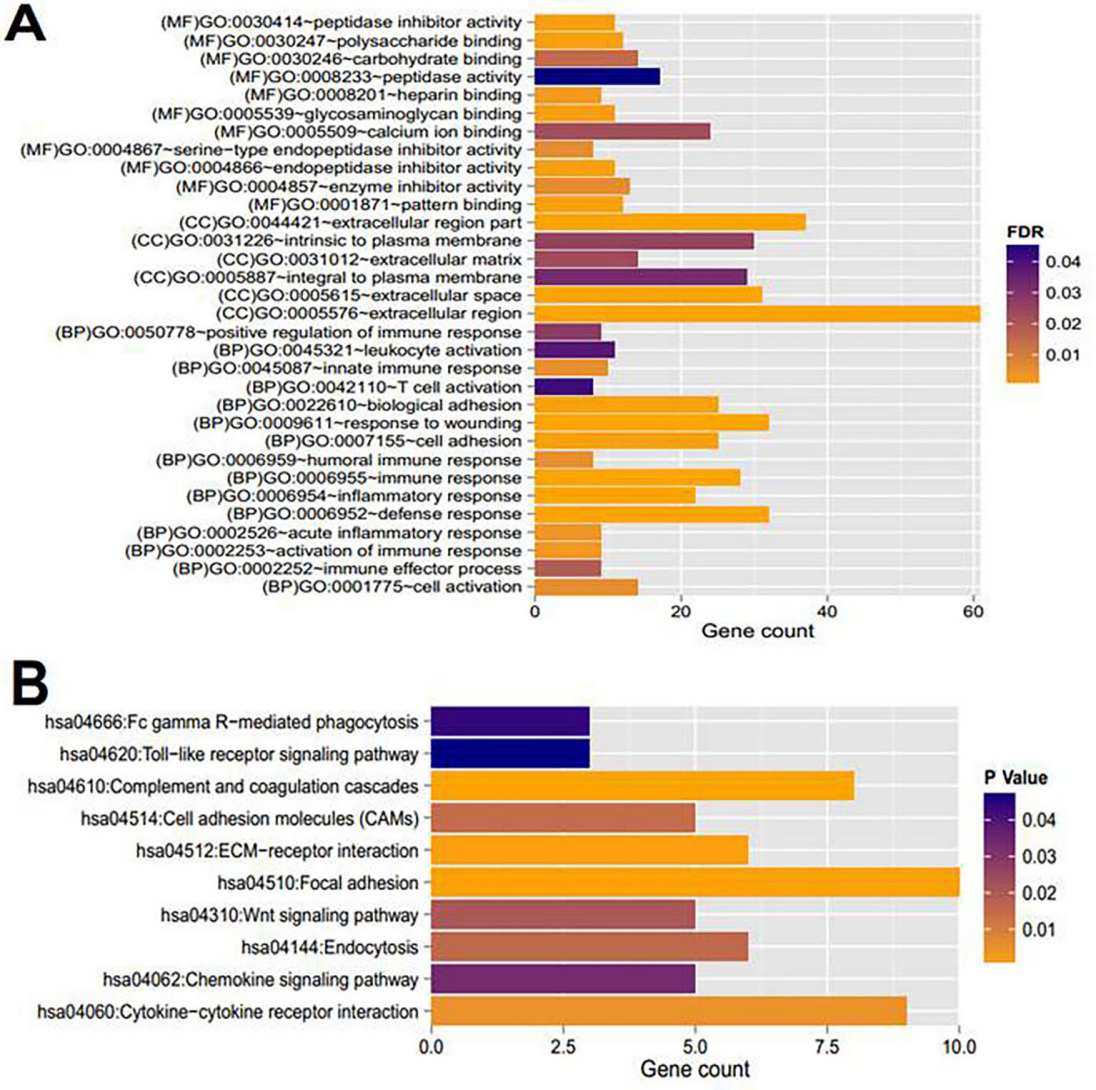
Enriched Gene Ontology functions (**A**) and KEGG pathways (**B**) of differentially expressed mRNAs and lncRNAs in the co-expression network. FDR: false discovery rate.

GO functional and KEGG pathway analyses using DAVID for differentially expressed mRNAs indicated 32 GO functions (15 biological processes, 6 cellular compartments, and 11 molecular functions) and 10 KEGG pathways were enriched (Table 3 and Figure 7). The differentially expressed mRNAs in the lncRNA-mRNA co-expression network were related to GO functions of wound response, defense response, immune response, and inflammatory response. Moreover, the enriched KEGG pathways included complement and coagulation cascades (hsa04610), ECM-receptor interaction (hsa04512), cytokine-cytokine receptor interaction (hsa04060), and cell adhesion molecules (hsa04514), etc.

**Table 3.** Enriched Gene Ontology (GO) functions and KEGG pathways of mRNAs in the co-expression network.

Category	Term	Count	P value
Biology process	GO: 0009611∼response to wounding	32	7.40E-14
	GO: 0006952∼defense response	32	3.77E-12
	GO: 0006954∼inflammatory response	22	1.90E-10
	GO: 0006955∼immune response	28	2.73E-08
	GO: 0022610∼biological adhesion	25	1.97E-06
	GO: 0007155∼cell adhesion	25	1.92E-06
	GO: 0002253∼activation of immune response	9	1.38E-05
	GO: 0002526∼acute inflammatory response	9	1.87E-05
	GO: 0006959∼humoral immune response	8	3.72E-05
	GO: 0045087∼innate immune response	10	3.42E-05
	GO: 0001775∼cell activation	14	3.19E-05
	GO: 0002252∼immune effector process	9	1.74E-04
	GO: 0050778∼positive regulation of immune response	9	2.97E-04
	GO: 0045321∼leukocyte activation	11	5.51E-04
	GO: 0042110∼T cell activation	8	6.86E-04
Cellular component	GO: 0005576∼extracellular region	61	2.57E-11
	GO: 0044421∼extracellular region part	37	2.49E-09
	GO: 0005615∼extracellular space	31	2.00E-09
	GO: 0031012∼extracellular matrix	14	4.48E-04
	GO: 0031226∼intrinsic to plasma membrane	30	6.13E-04
	GO: 0005887∼integral to plasma membrane	29	9.38E-04
Molecular function	GO: 0001871∼pattern binding	12	2.14E-06
	GO: 0030247∼polysaccharide binding	12	2.14E-06
	GO: 0004866∼endopeptidase inhibitor activity	11	8.62E-06
	GO: 0005539∼glycosaminoglycan binding	11	6.31E-06
	GO: 0030414∼peptidase inhibitor activity	11	1.38E-05
	GO: 0008201∼heparin binding	9	3.00E-05
	GO: 0004857∼enzyme inhibitor activity	13	8.93E-05
	GO: 0004867∼serine-type endopeptidase inhibitor activity	8	1.09E-04
	GO: 0030246∼carbohydrate binding	14	3.03E-04
	GO: 0005509∼calcium ion binding	24	5.12E-04
	GO: 0008233∼peptidase activity	17	0.001293
KEGG pathway	*hsa04610: Complement and coagulation cascades	8	8.31E-06
	*hsa04510: Focal adhesion	10	0.000368
	hsa04512: ECM-receptor interaction	6	0.000945
	*hsa04060: Cytokine-cytokine receptor interaction	9	0.004923
	hsa04514: Cell adhesion molecules (CAMs)	5	0.014839
	*hsa04144: Endocytosis	6	0.015668
	hsa04310: Wnt signaling pathway	5	0.020687
	*hsa04062: Chemokine signaling pathway	5	0.033087
	*hsa04666: Fc gamma R-mediated phagocytosis	3	0.043681
	*hsa04620: Toll-like receptor signaling pathway	3	0.046832

### KEGG pathway network further revealed the prognostic lncRNA biomarkers related to CKD

**Figure 8.**
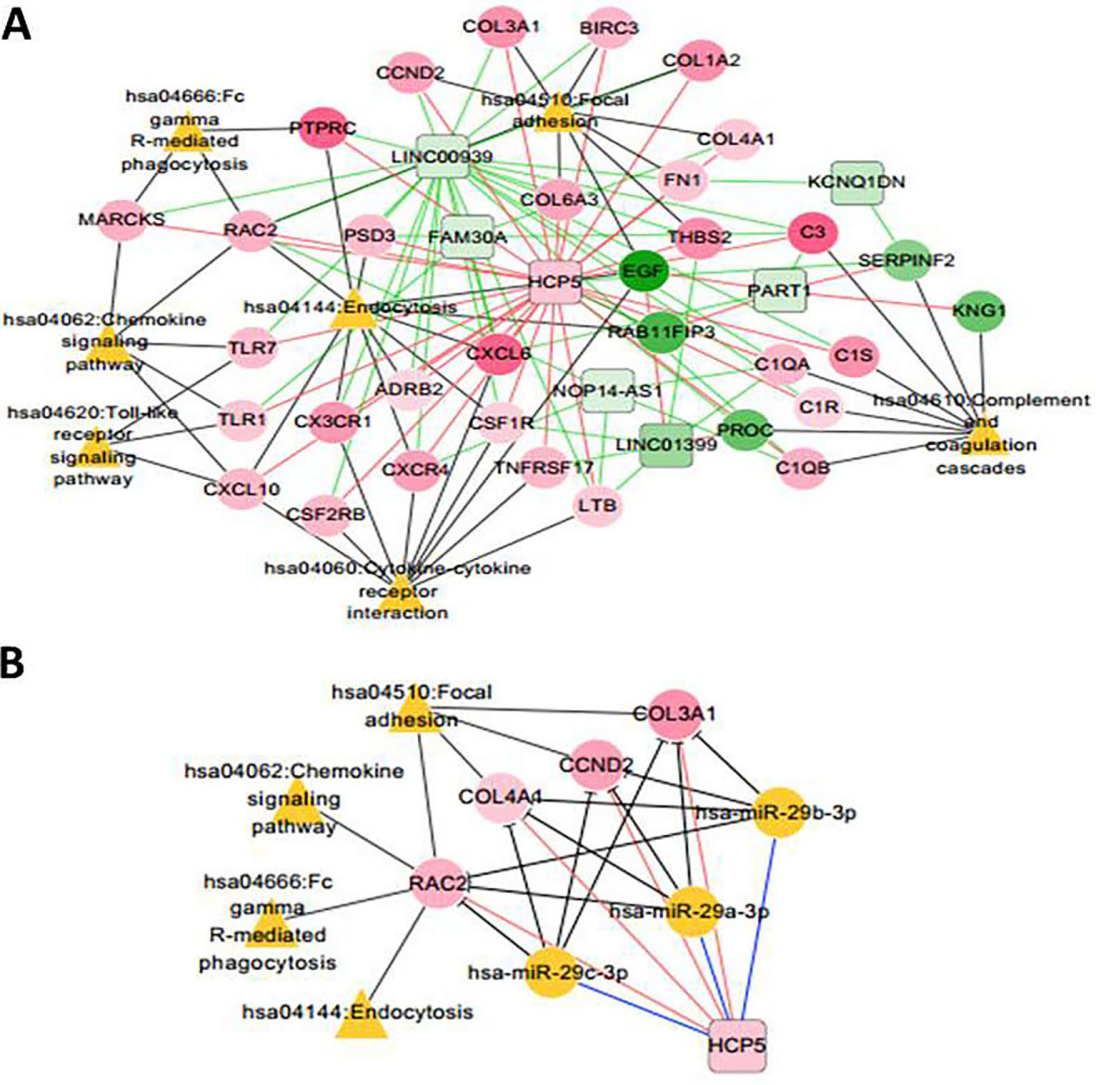
A lncRNA-mRNA-pathway network (**A**) and a lncRNA-miRNA-mRNA-pathway network (**B**) related to chronic kidney disease (CKD). Green lines represent negative correlations; pink lines represent positive correlations; black lines represent gene connection with KEGG pathways. Squares indicate lncRNA; circles indicate mRNA; yellow circles indicate miRNA; triangles indicate pathways. The node change from green to pink indicates that log fold change changed from negative to positive.

**Figure 9.**
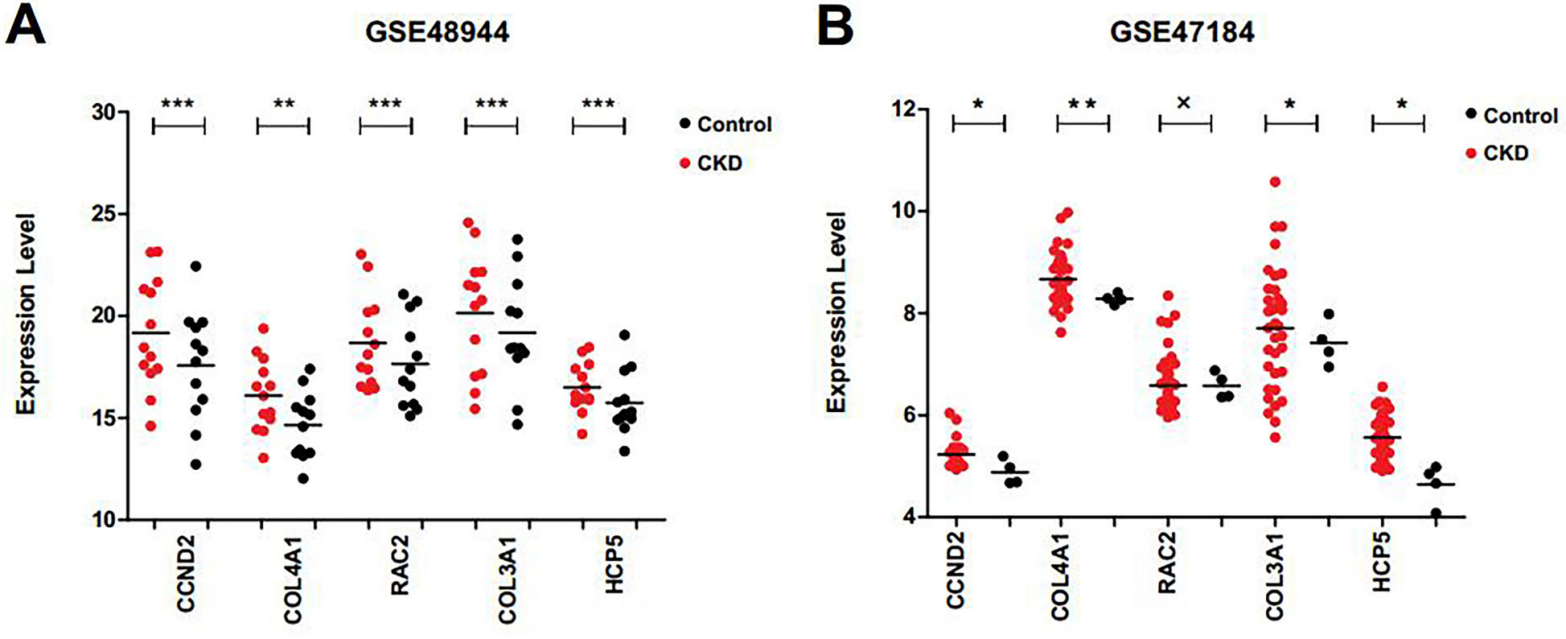
Expression level of *CCND2*, *COL3A1*, *COL4A1*, *RAC2*, and *HCP5* in GSE48944 and GSE47184 in controls and in chronic kidney disease (CKD) patients. *P<0.05; **P<0.01; ***P<0.005. x indicates not significant. Statistical analyses were performed with the *t*-test.

Using the keyword “Chronic Kidney Disease”, 175 KEGG pathways related to CKD were searched with CTD, as shown in Table S6. These pathways were compared with KEGG enriched pathways in the co-expression network of differentially expressed mRNAs and lncRNAs, and 7 overlapping pathways were obtained (Table 3, indicated with *). A lncRNA-mRNA-pathway network related to CKD was constructed using these overlapping pathways, as shown in Figure 8A. The miRNAs related to 7 lncRNAs in the network were searched using StarBase Version 2.0 database, and 24 lncRNA-miRNA linking relations were found, involving two lncRNAs of HCP5 and NOP14-AS1 (Table S7). Next, a lncRNA-miRNA-mRNA-pathway network was constructed by connecting the has-miR-29a/b/c, HCP5, and 4 KEGG pathways. As shown in Figure 8B, the four genes of *CCND2*, *COL3A1*, *COL4A1*, and *RAC2*, positively related with *HCP5*, were found to participate in four KEGG pathways directly related to CKD. As shown in Figure 9, *CCND2*, *COL3A1*, *COL4A1*, *RAC2*, and *HCP5* were significantly upregulated in GSE48944 and GSE47184 (besides *RAC2* in GSE47184).

## Discussion

The results of this study indicated that genes with similar functions within modules could contribute to the risk of CKD in a co-expression manner, and the modules with different functions could be regulated synergistically.

In order to identify potential critical lncRNAs and genes associated with CKD, we focused our attention on hub lncRNAs and genes in the lncRNA-miRNA-mRNA-pathway network. Downregulated has-miR-29a/b/c was reported to be related to CKD directly (22). *NOP14-AS1* (NOP14 antisense RNA 1) is affiliated with the non-coding RNA class. Diseases associated with *NOP14-AS1* include astrocytoma. Previous research identified that *NOP14-AS1* was strongly regulated by DNA damaging agents and significantly negatively correlated with its sense gene *NOP14* in a p53-dependent manner. Hence, sense-antisense pair of *NOP14-AS1/NOP14* might be involved in the progression of CKD upon DNA damage induction. The long non-coding RNA-HLA complex P5 (HCP5) has been found to be overexpressed in follicular thyroid carcinoma, which functions as a competing endogenous RNA and acts as a sponge for several miRNAs (23). The lncRNA-miRNA-mRNA-pathway network constructed indicated that *HCP5* might be regulatory genes associated with the progression of CKD via the genes of *CCND2*, *COL3A1*, *COL4A1*, and *RAC2*. The *CCND2* gene encodes a protein G1/S-specific cyclin-D2 in humans, which belongs to the highly conserved cyclin family. Cyclins function as regulators of cyclin-dependent kinases. High level expression of *CCND2* gene was observed in ovarian and testicular tumors (24). *COL3A1* gene encodes a protein of collagen alpha-1(III) chain, a precursor to collagen III, which is found in extensible connective tissues, frequently in association with type I collagen (25). The *COL4A1* gene contains one of 27 SNPs associated with increased risk of coronary artery disease (26). *RAC2* gene encodes a small (∼21 kDa) signaling G protein, a member of the Rac subfamily of the Rho family of GTPases, which has been shown to interact with nitric oxide synthase 2A (27). Thus, these are important genes for CKD.

In summary, this work revealed that lncRNA of *NOP14-AS1* and *HCP5* might be potential genetic biomarkers for the progression of CKD. They may function via the genes of *CCND2*, *COL3A1*, *COL4A1*, and *RAC2*, and the pathways of complement and coagulation cascades (hsa04610), ECM-receptor interaction (hsa04512), cytokine-cytokine receptor interaction (hsa04060), and cell adhesion molecules (hsa04514), etc. Although a comprehensive analysis was performed, there were a number of limitations in the work. Functional validation was not done for the hub lncRNAs and mRNAs obtained. Further investigations are required with substantial experiments. Nevertheless, this work provides novel insights into the occurrence and progression of CKD.

## Supplementary Material

Click here to view [xls].
